# Modeling the Anti-Adhesive Role of Punicalagin Against Listeria Monocytogenes from the Analysis of the Interaction Between Internalin A and E-Cadherin

**DOI:** 10.3390/ijms26157327

**Published:** 2025-07-29

**Authors:** Lorenzo Pedroni, Sergio Ghidini, Javier Vázquez, Francisco Javier Luque, Luca Dellafiora

**Affiliations:** 1 Department of Food and Drug, University of Parma, 43124 Parma, Italy; lorenzo.pedroni@unipr.it; 2 Department of Veterinary Medicine and Animal Sciences, University of Milan, 20122 Milan, Italy; sergio.ghidini@unimi.it; 3 Departament de Nutrició, Ciències de l’Alimentació i Gastronomia, Facultat de Farmàcia i Ciències de l’Alimentació, Institut de Química Teòrica i Computacional (IQTCUB) and Institut de Biomedicina (IBUB), Universitat de Barcelona, 08021 Santa Coloma de Gramenet, Spain; javier.vazquez@pharmacelera.com (J.V.); fjluque@ub.edu (F.J.L.); 4 Pharmacelera, Parc Científic de Barcelona (PCB), Baldiri Reixac 4-8, 08028 Barcelona, Spain

**Keywords:** *Listeria monocytogenes*, internalin A, E-cadherin, molecular modeling, antimicrobial natural compounds, food bioactives

## Abstract

*Listeria monocytogenes* poses health threats due to its resilience and potential to cause severe infections, especially in vulnerable populations. Plant extracts and/or phytocomplexes have demonstrated the capability of natural compounds in mitigating *L. monocytogenes* virulence. Here we explored the suitability of a computational pipeline envisioned to identify the molecular determinants for the recognition between the bacterial protein internalin A (InlA) and the human E-cadherin (Ecad), which is the first step leading to internalization. This pipeline consists of molecular docking and extended atomistic molecular dynamics simulations to identify key interaction clusters between InlA and Ecad. It exploits this information in the screening of chemical libraries of natural compounds that might competitively interact with InIA and hence impede the formation of the InIA–Ecad complex. This strategy was effective in providing a molecular model for the anti-adhesive activity of punicalagin and disclosed two natural phenolic compounds with a similar interaction pattern. Besides elucidating key aspects of the mutual recognition between InIA and Ecad, this study provides a molecular basis about the mechanistic underpinnings of the anti-adhesive action of punicalagin that enable application against *L. monocytogenes*.

## 1. Introduction

Microbiological hazards in food and feed production and processing, primarily associated with *Salmonella enterica*, *Listeria monocytogenes* (LM), and *Cronobacter sakazakii*, is a major concern for human health [1]. Antibiotics are the only available treatment against infections caused by foodborne pathogens. However, the increase in antibiotic resistance and the side effects of synthetic compounds make it necessary to search for alternative therapies, including the usage of natural products [2,3,4]. Indeed, besides their antimicrobial properties, they are promising starting points for the development of new antimicrobial agents due to their low toxicity, high bioavailability, and excellent bioactivity [5,6,7].

Food exposure is the main culprit of infections by LM, which is a ubiquitous Gram-positive and facultative anaerobic bacterium that can be found everywhere, ranging from soil to water to food or feed, and can replicate even at refrigeration temperatures (from 0 °C) and under a broad pH range (roughly from 4 to 9) [8,9]. This explains why many steps of food processing, especially concerning dairy products, vegetables, or ready-to-eat products, can be contaminated by LM, leading to listeriosis in animals and humans [10,11]. Although the number of *Listeria*-related infections is lower compared to other foodborne pathogens, the global burden of listeriosis remains significative due to the high mortality rate (up to 30%), especially in people belonging to the so-called YOPI group (young, old, pregnant, and immunosuppressed) [12,13].

Searching for innovative strategies with which to mitigate LM infections makes it necessary to gain detailed knowledge of the molecular mechanisms implicated in cell invasion and intracellular growth of the pathogen. LM infection begins with the invasion of human intestinal cells where it crosses their membrane by inducing its own endocytosis [14,15]. This process is primarily mediated by internalins (Inl), which are a class of proteins exposed on the pathogen’s outer membrane. Among the five main representatives, namely InlA/B/C/F/P, InlA/B/F facilitate the internalization through interaction with E- cadherin (Ecad), which is a surface protein in human (and mammalian) cells [14,16]. Of note, the InlA-driven internalization promotes quick LM transcytosis across the intestinal epithelium, possibly leading to severe infective outcomes such as meningoencephalitis or, in pregnant women, fetal–placental infection [15,17].

Crystallographic studies solved the structure of the InlA–Ecad complex, thus providing valuable information with which to identify key interactions between InIA and E-cad [18,19,20]. InlA (460 residues) presents α/β folding characterized by a leucine-rich repeat (LRR) domain adjacent to an inter-repeat domain resembling an immunoglobulin fold (Figure S1, Supplementary Materials). The LRR domain binds the Ecad extracellular domain, leading to LM internalization [21]. Remarkably, the occurrence of LM strains not harmful to humans has been associated with InlA mutations that impede the interaction with cadherins, thus preventing effective cell invasion [22,23]. In this regard, in silico studies identified key InlA mutations that disfavored the interaction with Ecad [24]. Furthermore, LM strains pathogenic to humans could not infect mice, largely due to differences in some residues on the mouse Ecad [25,26,27]. Overall, finding effective strategies to prevent the interaction between InlA and Ecad could serve as a promising strategy to hamper cell invasion, possibly mitigating LM virulence.

Following this strategy, previous studies examined the effect of short peptides on inhibiting the invasion of target cells by LM using molecular modeling and cell invasion assays [28,29]. Sahu et al. demonstrated that a short peptide comprising residues 1–30 from human E-cadherin inhibited the invasion of Caco-2 cells by LM [29]. Importantly, the anti-invasion activity was eliminated for the corresponding mouse peptide, where residue P16 in human Ecad is replaced by E, thus suggesting that the human peptide interferes with the formation of the complex between InlA and E-cadherin. These findings support the feasibility of searching for alternative compounds that might exhibit similar anti-adhesive effects.

There is abundant evidence demonstrating the anti-LM activity of pomegranate extracts [30,31,32,33,34,35,36], suggesting that phenolic compounds and specifically punicalagin, which is the major component of the tannin-rich extract [31,32,37], may be responsible for the antimicrobial activity. Indeed, Gosset-Erard et al. demonstrated that pure punicalagin exhibits antibacterial activity against various yeast strains and both Gram-negative and Gram-positive bacteria (minimal inhibitory concentrations ranging from 0.3 to 1.2 mg/mL) [38]. Furthermore, previous studies demonstrated that a pomegranate punicalagin-rich (64.2%, g/g) fraction reduced, in a dose-dependent manner, LM adhesion by ca. 50% at a concentration of 1.25 mg/mL [31]. However, to the best of our knowledge, there is no structural basis for the anti-adhesive activity of punicalagin.

With adhesion being an early and crucial step in LM pathogenesis, we hypothesize that the anti-adhesive activity of punicalagin-rich extracts may, at least in part, reflect the ability to disrupt the interaction between InlA and Ecad. In this context, we aimed to fill this knowledge gap by addressing three complementary objectives. First, by exploiting the structural information available for the InlA–Ecad, we aim to explore the potential interaction of punicalagin with InlA and characterize the structural and energetic determinants of InlA–Ecad recognition. Second, given the structural complexity of punicalagin, we aim to exploit the knowledge about the key features that mediate the interaction with InlA to identify putative smaller compounds by screening a curated library of natural compounds. Specifically, the molecular determinants involved in InlA–Ecad recognition and binding were used to guide the screening of chemical libraries of natural compounds, mostly present in plant extracts and phytocomplexes that might impair the formation of the InlA–Ecad complex. To this end, a virtual library of ~600,000 natural compounds was compiled from three databases, namely COCONUT (https://coconut.naturalproducts.net/ (accessed on 20 March 2024)) [39], LOTUS (https://lotus.naturalproducts.net/documentation (accessed on 20 March 2024)) [40], and FOODB (https://foodb.ca/ (accessed on 20 March 2024)), and narrowed down to ~5000 compounds through a multi-tier filtering approach. This approach allowed us to retrieve a few polyphenolic compounds, including punicalagin, whose recognition is assisted by key interaction motifs present in InIA. Overall, the strategy outlined here proved effective for exploring food-grade natural compounds with anti-adhesive activity.

## 2. Results and Discussion

### 2.1. Characterization of Recognition Motifs at the InlA–Ecad Interface

We applied a computational MD-based pipeline to gain structural insights into InlA–Ecad interaction and identify key interactions that mediate the recognition and binding between InIA and Ecad. To this end, three replicas of 1 μs long MD simulations were run to investigate InlA–E-cad complex stability and to identify key residues contributing to the intermolecular interaction.

**Figure 1:**
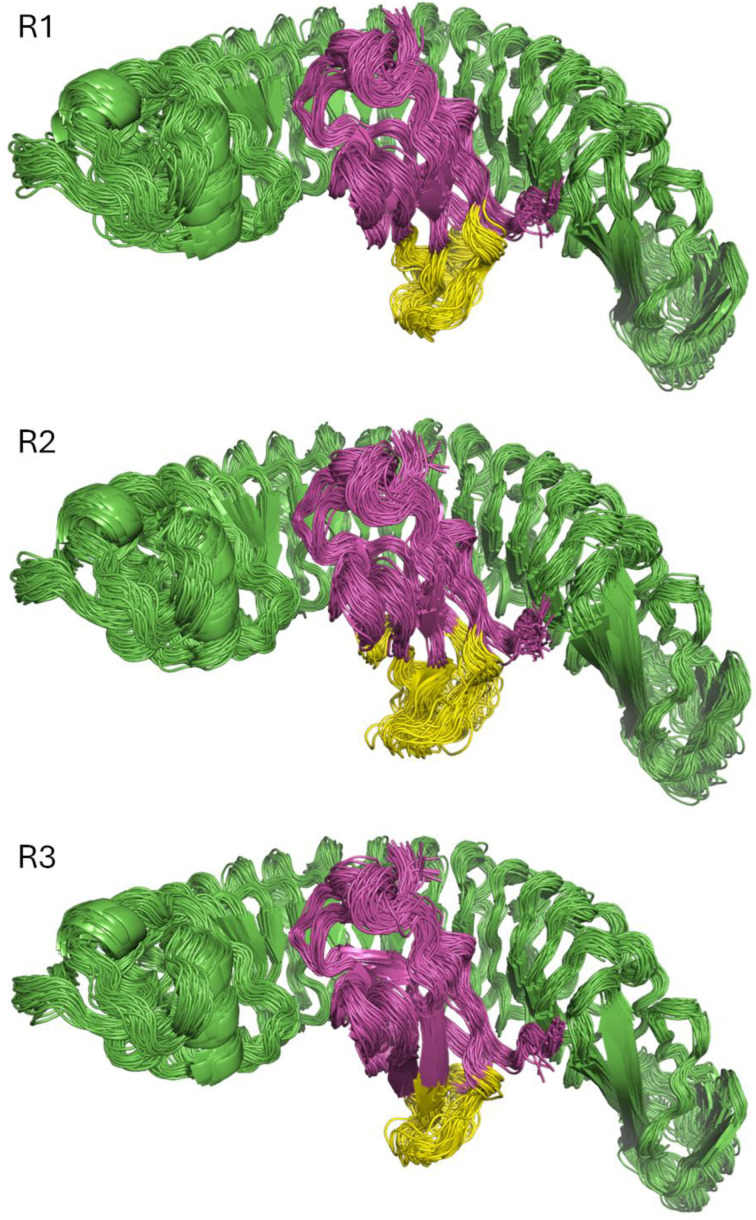
Collection of 50 snapshots (1 every 20 ns) of the 1 μs long MD simulations replicas (R1, R2, and R3). InlA is represented by the green cartoon and Ecad by the magenta cartoon, with the mobile loop in yellow.

Apart from the N- and C-terminal regions, which were expected to be more mobile in the two proteins, the overall complex was highly stable, especially on the InlA side, as noted in the superposition of snapshots taken regularly along the three trajectories (Figure 1). On the other hand, Ecad showed a loop (shown in yellow in Figure 1) that was consistently less stable, especially in the second replica. This was expected since this region is the most exposed to the solvent, and it is not interacting with InlA. Moreover, the root mean squared deviation (RMSD) of both InlA and Ecad confirmed the structural stability of the complex for the three independent MD simulations, as noted in values comprised between 1.0 and 1.5 Å for InlA and 1.5 and 2.0 Å for Ecad (Figure S2, Supplementary Materials).

The presence of specific interaction motifs at the InlA–Ecad interface was investigated for an ensemble of snapshots collected every 20 ns from the MD simulations, which were inspected with the aim of finding conserved intermolecular contacts in the three replicas. This analysis allowed us to identify four interaction clusters between InIA and E-Cad, along with a cavity between the third and fourth clusters (noteworthily, this cavity might be a putative binding site of ligands preventing/impairing InlA–Ecad interaction, although this strategy has not been further considered in this study).

**Figure 2:**
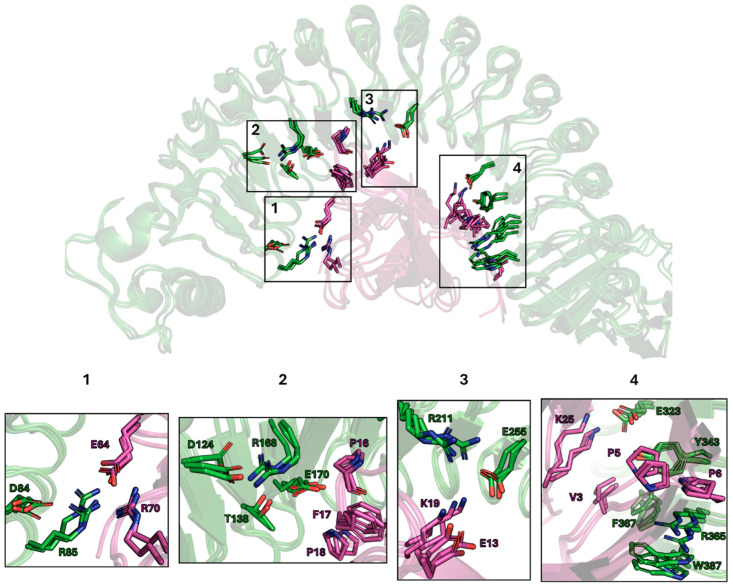
Inla (green)—Ecad (magenta) representation of the three replicas with most represented interacting residues reported as sticks. The four identified clusters of interaction are numbered 1 to 4 and embedded within black squares. The residues are numbered according to the PDB ID 1O6S. Of note, the visible gap between clusters 3 and 4 is occupied by a conserved cavity on the InlA–Ecad interface.

The first three clusters encompass more than half of the InlA–Ecad interaction surface (Figure 2). Indeed, based on the analysis of the contact surface area (CSA) between InlA and E-cad, it was noticed how the region embedded by the first three clusters contributes to roughly 50% (~700 Å^2^) of the total InlA-Ecad CSA (~1400 Å^2^). The first three clusters were mainly characterized by the presence of arginine and aspartate residues, conferring a typical polar environment, and the fourth one was mainly hydrophobic (Figure 2). From a structural point of view, clusters 1–3 are located grouped in the same region of the InIA surface, thus providing multiple contacts that can be exploited to promote cooperativity in the formation of multiple interactions with putative binding partners. In contrast, cluster 4 is placed in a separate area of the InIA surface, distal to clusters 1–3, which makes it more challenging to be targeted simultaneously by small- and medium-sized natural compounds. Therefore, the interaction with clusters 1–3 was prioritized in the screening of the chemical library.

Another reason to prefer targeting the region covering clusters 1–3 is related to the differences between human and murine Ecad. As shown in previous studies, mice are resistant to LM infection since their Ecad brings some different residues exposed to the interface with InlA [25,26]. Accordingly, we performed additional MD simulations of the murine Ecad–InlA complex (Figure S3) following the protocol adopted for the human complex to examine the effect of the mutated residues on the formation of the complex. Of note, some changes correspond to conserved mutations or affect solvent-exposed residues not involved in the interaction with Ecad; others imply more relevant changes, such as P16→E. Accordingly, the mutated residues were visually inspected to avoid steric clashes or improper structural arrangements. Furthermore, the simulations performed for the murine Ecad were subjected to the same setup adopted for the human protein through the sequential energy minimization, thermalization, and equilibration steps prior to the production of the trajectory (see Section 3.2).

Although the structural stability of the murine complex is comparable with the results obtained for the human complex within the time scale of the MD simulations (see the RMSD profiles shown in Figure S3), the per-residue free energy decomposition analysis of the energetic contribution between InIA and both murine and human Ecad disclosed significant differences in the contribution of specific residues to the energetic stability of the complex (Table 1). Such differences in the interaction energy primarily affect P16 and E64 in human Ecad, which are replaced by E and Q, respectively, in murine Ecad. These results confirmed the importance of P16, which is embedded between the second and third interaction clusters (Figure 2), in driving a proper interaction with InlA, in agreement with previous experimental and computational results [26,27,29]. On the other hand, E64 is placed in cluster 1 (Figure 2).

The structural stability of these interaction clusters was also assessed through MD simulations of the apo form of InIA, which was generated upon the removal of Ecad from the complex, since the geometrical arrangement of the clusters may be significantly altered in the absence of Ecad. To this end, we performed three independent MD simulations (1 μs each) of InIA. The structural fold of the protein remained stable, as noted in the superposition of the snapshots taken along the trajectories, and the RMSD profiles obtained for the protein backbone, which correspond to average values close to 2.0 Å (Figure S4, Supplementary Materials). Notably, a slightly larger value (RMSD of 2.4 Å) was determined for the set of residues included in clusters 1–3.

Overall, these results give support to the strategy of exploiting these interaction motifs in the search for compounds valuable to preventing LM adhesion.

### 2.2. Docking-Based Screening and Analysis of InlA-Ligand Stability

The library of curated natural compounds compiled from COCONUT, LOTUS, and FOODB (last databases access 20 March 2024) was filtered based on molecular weight, logP, rotatable bonds, and molecular length (see Section 3.1.2), yielding a final subset of 4986 compounds. Next, they were screened using the docking-based procedure implemented in Glide, which combined HTVS and subsequently SP scoring functions (see Section 3.3. for further details) [41]. The first 500 poses were visually inspected on PyMol (v. 2.3) to identify the most promising compounds, whose binding mode was finally refined using MD simulations of the corresponding InIA–ligand complex.

**Figure 3:**
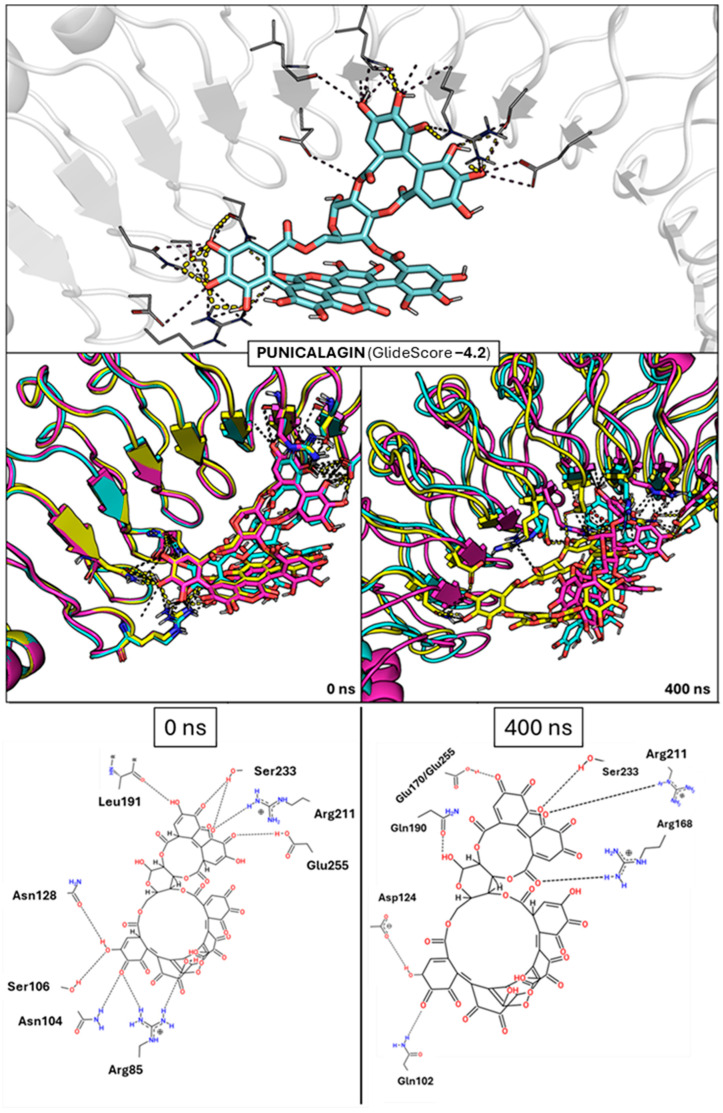
On the top, architecture of binding of punicalagin along with the docking score. InlA is represented by the white transparent cartoon, while the interacting residues are white lines. Punicalagin is represented by cyan sticks with polar interactions represented by yellow dashed lines. In the middle panel, focus is on the first (0 ns) and last (400 ns) frames of the three MD simulation replicas between InlA and punicalagin. Punicalagin and the interacting residues are represented as sticks and differently colored depending on the replica (magenta, cyan, and yellow) with polar interactions represented as dashed lines. On the bottom, 2D diagrams of the average contacts at times 0 ns and 400 ns for the three MD independent replicas of the interaction between punicalagin and InlA, with black dashed lines indicating hydrogen bonds.

**Figure 4:**
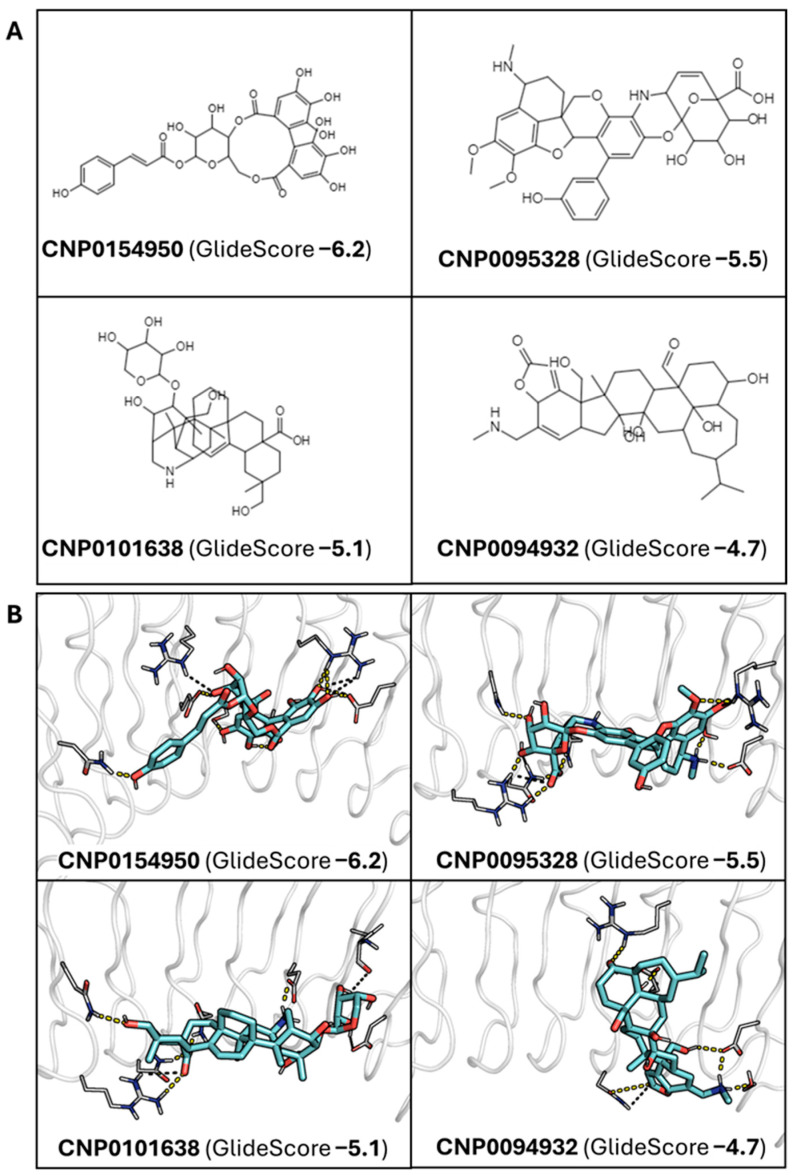
(**A**) Chemical sketches of the selected compounds from the docking-based screening procedure along with their database ID and docking score. (**B**) Architecture of the binding of each compound along with their database ID and docking score. InlA is represented by the white transparent cartoon, while interacting residues are white lines. The ligands are represented by cyan sticks. Polar interactions are represented as yellow dashed lines.

This procedure allowed us to retrieve punicalagin (GlideScore: –4.2 kcal/mol; Figure 3) as well as four polyphenolic molecules of lower molecular weight, with docking scores ranging from −6.2 to −4.7 kcal/mol (Figure 4A): CNP0154950 (GlideScore −6.2), CNP0095328 (GlideScore −5.5), CNP0101638 (GlideScore −5.1), and CNP0094932 (GlideSCore −4.7). Compared to punicalagin, these compounds exhibit a high similarity in the molecular fields that reflect the 3D distribution of hydrophobic/philic properties determined from the quantum mechanically derived HyPhar descriptors [42,43], which provide information about the 3D atomic distribution pattern of the n-octanol/water partition coefficient (see Figure S5, Supplementary Materials). Thus, the similarity index (Tversky metrics) determined for the pairwise overlay with punicalagin ranges from 0.80 for CNP0101638 to 0.68 for CNP0095328. This supports the feasibility of these compounds to establish a similar pattern of noncovalent interactions.

Visual inspection of the docked poses revealed certain common interaction patterns for some of the compounds (Figure 4B). CNP0095328 and CNP0101638 were both engaged in salt bridges with R85, which forms a salt bridge with E64 of human Ecad in the InIA–Ecad complex, and either D213 (CNP0095328) or E170 (CNP0101638), which in turn forms a hydrogen bond with the backbone NH of F17 of human Ecad in the InIA–Ecad complex. Moreover, regarding other polar contacts, these two compounds along with CNP0154950 kept comparable patterns of interaction. Furthermore, these polar contacts were supplemented with a hydrophobic interaction with F150, which interacts with residues F17 and P18 of Ecad in the InIA–Ecad complex. In contrast, a distinct interaction pattern was found for CNP0094932 (Figure 4B). It is worth noting, however, that caution is necessary to not overemphasize the significance of these interaction patterns due to the use of a rigid docking procedure, which may affect the mutual adaptability of both ligand and target protein.

### 2.3. Analysis of MD Simulations of the InlA–Ligand Complexes

Considering the uncertainties of docking calculations, the reliability of these interactions was examined from the analysis of MD simulations performed for the distinct InIA–ligand complexes. However, due to the anti-adhesive activity of punicalagin (see above), three independent 400 ns MD simulations were initially performed for the InIA–punicalagin complex (i) to explore the potential of punicalagin to stably interact with key InlA residues and (ii) to validate the suitability of the in silico pipeline to verify meaningful interaction patterns within this complex system.

The results of these simulations supported both assumptions. First, punicalagin exhibited reasonably stable binding across the three replicas (Figure 3). Indeed, stable RMSD profiles were obtained for the three simulations, with average RMSD values varying between 1.0 and 2.0 Å (see Figure S6, Supplementary Materials). Certain structural rearrangements that mainly affected the chemical moiety of punicalagin that interacts with cluster 1 were observed. This can be attributed to the larger solvent exposure of this area, since thermal fluctuations may favor the interaction of water molecules instead of maintaining the interactions with punicalagin. However, this did not compromise the compound’s global binding behavior, as punicalagin consistently reinforced interactions with clusters 2 and 3. Thus, the main interactions involved residues R211, S233, and E255, and they were consistently observed across the three MD independent replicas (Table S1, Supplementary Materials). Interestingly, R211 and E255 are part of the third conserved interaction cluster identified in the InlA–Ecad complex (Figure 2). Of note, these residues occupy the region usually contacted by P16 in human Ecad, which is key for the formation of the InlA–Ecad complex. These findings suggest that punicalagin may exert anti-adhesive/invasive activity, mainly interfering with these key residues embedded within clusters 2 and 3. On the other hand, the analysis of the interaction energy performed with the MMPBSA method led to an estimated interaction of approximately −18 kcal/mol. As expected, this is weaker than the InlA–Ecad native interaction (ca. −38 kcal/mol). Although the interaction energies have to be considered with caution due to the approximations included in MMPBSA calculations, our results reflect the wider protein–protein interface due to the multivalent interaction of Ecad with clusters 1–4, whereas the interaction with clusters 1–3 roughly accounts for half the protein–protein interface (see above).

**Figure 5:**
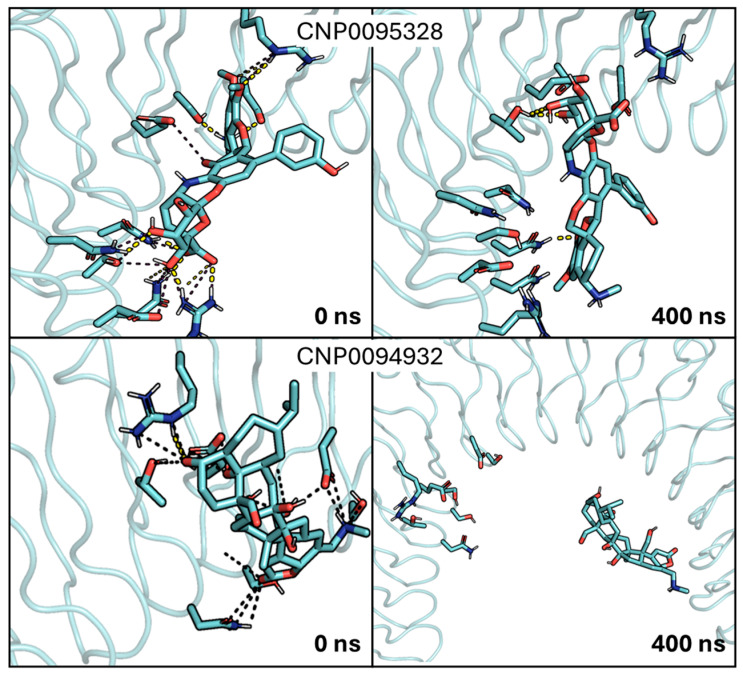
Focus on the first (0 ns) and last (400 ns) frames of the MD simulations between InlA and the two discarded ligands. Both ligands, CNP0095328 and CNP0094932, are represented by cyan sticks, with polar interactions reported as dashed lines. In the upper part it can be appreciated how the initial pattern of interaction between InlA and CNP0095328 is not maintained, while in the lower part the complete detachment of CNP0094932 from InlA can be observed.

In light of these results, we examined the stability of the binding mode of the four candidates by running additional MD simulations (400 ns). Regarding CNP0094932, it showed a clear instability along the MD simulation, eventually detaching from InlA (Figure 5 and Figure S8, Supplementary Materials). CNP0095328 lost the first pattern of interactions that justified its inclusion among the putative candidates, partially detaching from InlA (Figure 5 and Figure S8, Supplementary Materials). For these reasons, they were both discarded. Of note, this highlights certain limitations of docking calculations—especially when using a rigid body approach—that cannot capture the dynamic behavior of ligands within the binding site and/or account for solvent interactions. Although docking was useful for the initial screening, MD simulations were essential for refining the selection of the compounds by revealing instabilities not evident upon inspection of the static poses, as noted in previous studies [44,45].

**Figure 6:**
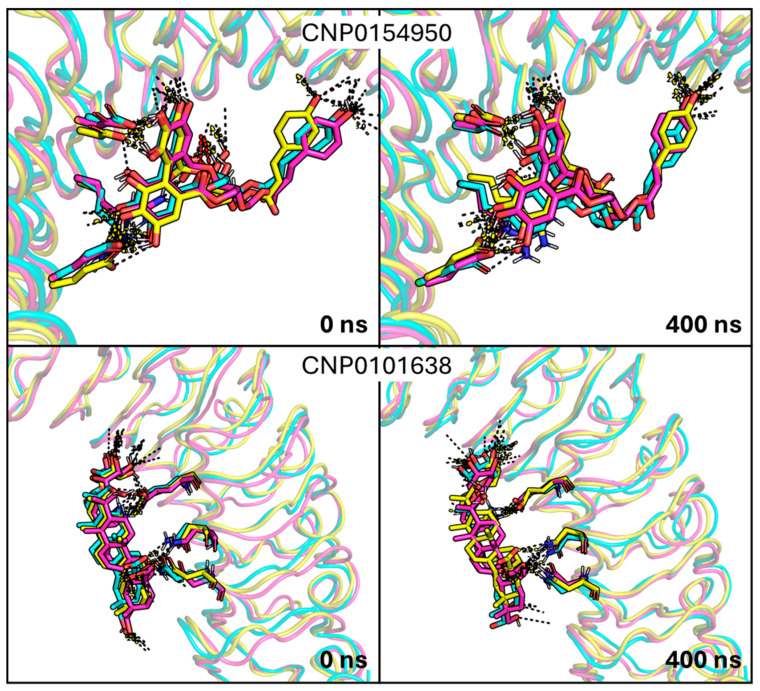
Focus on the first (0 ns) and last (400 ns) frames of the three MD simulations replicas between InlA and the two promising ligands. In the upper part, CNP0154950 and the interacting residues are represented by sticks and are differently colored depending on the replica (magenta, cyan, and yellow), with polar interactions reported as dashed lines. In the lower part, CNP0101638 and the interacting residues are represented by sticks and are differently colored depending on the replica (magenta, cyan, and yellow), with polar interactions reported as dashed lines.

In contrast, compounds CNP0154950 and CNP0101638 showed a stable binding mode, which was subsequently confirmed by running two additional MD simulations (Figure 6 and Figure S9, Supplementary Materials). The results of these independent simulations exhibited comparable behavior in all of the replicas, thus confirming the stability of the binding mode (Figure S7, Supplementary Materials).

**Figure 7:**
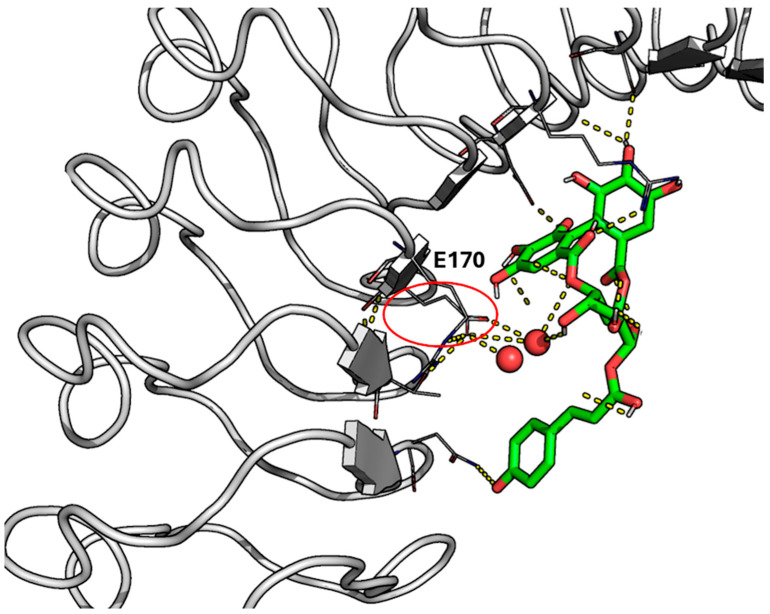
Focus on the “hole” formed between CNP0154950 and InlA. InlA is reported as a white cartoon, the ligand as green sticks, and the interacting InlA residues as white lines with polar interaction reported as yellow dashed lines. Of note, E170 is embedded in the red circle. Water is reported as a red sphere. It can be appreciated how water molecules act as bridges to keep a hydrogen-bonding interaction between a hydroxylate group belonging to the ligand and InlA E170.

Figure 6 and Figure S9 (Supplementary Materials) show that the pattern of interactions found for CNP0154950 and CNP0101638 is preserved along the whole simulation, thus supporting the overall stability afforded by the cooperativity of the multiple interactions formed with InlA. As shown in Figure S7 (Supplementary Materials), the RMSD profile obtained for the simulations of the complex with CNP0154950 was stable over 400 ns for two distinct MD simulation replicas. Of note, one replica showed some small short-lasting readjustments within the first half of the simulation, but it reached a stable state keeping a good amount of hydrogen bonds till the end of simulation. Interestingly, shortly after the beginning of the MD simulations, we noticed the formation of a “hole” next to the ligand tyrosine-moiety, which was filled by water molecules acting as bridge to maintain a hydrogen bond between a hydroxyl group from CNP0154950 and an E170 of InlA (Figure 7). The visual inspection of the ensemble, based on snapshots extracted every 10 ns from each independent MD replica, revealed that CNP0154950 maintained a direct hydrogen bond with residue E170 of InlA for ~34% of the simulation time (400 ns), while in the remaining ~66% of the trajectory the interaction was mediated by a bridging water molecule. Beyond this interaction, this compound maintained hydrogen bonds with D213, E255, and N128 for more than half of the MD simulation time. Other significant interactions included hydrogen bonds with S192 and R211, which were maintained for more than a third of the MD simulations. The analysis of the interaction energy performed with MMPBSA led to an estimated value of approximately −14 kcal/mol, which is slightly lower than that obtained for punicalagin.

CNP0101638 behaved similarly, maintaining a comparable stable binding mode, as shown in the RMSD profile in Figure S7 (Supplementary Materials). While the salt bridge with R85 was not consistently preserved, the ligand formed hydrogen bonds with N107 and R211 and a salt bridge with E170 for roughly half of the MD simulation time. Additionally, CNP0101638 maintained a hydrogen bond with N128 for more than one-fourth of the MD simulations. Moreover, looking at the MMPBSA analysis, the interaction energy of CNP0101638 (−17 kcal/mol) is comparable to the value estimated for punicalagin.

Overall, the computational protocol discussed here was able to retrieve punicalagin as a compound able to prevent the formation of the complex between InIA and Ecad. This finding agrees with the experimentally determined anti-adhesion activity found against LM infections. The present results provide a molecular basis with which to relate the anti-adhesive activity of punicalagin to the formation of multiple interactions with InIA, primarily involving residues in clusters 2 and 3, which encompass P16, whose mutation to E in the murine Ecad prevents the internalization of LM. Compared to the InIA–Ecad complex, the interaction energy of the punicalagin–InIA complex (ca. −18 kcal/mol) represents ca. 50% of the protein–protein interaction energy (ca. −38 kcal/mol), reflecting primarily the interaction with clusters 2 and 3. Clearly, the multivalent contacts formed by Ecad with clusters 1–4 lead to the larger stabilization of the fully assembled InIA–Ecad complex. However, the access of punicalagin to the interaction sites in InIA may be kinetically favored due to the ability of the polyphenolic structure to form multiple interactions, and to the smaller size relative to Ecad. Therefore, the prior binding of punicalagin to clusters 2 and 3 would impose a drastic alteration in the exposed surface of InIA, which would prevent the formation of the thermodynamically favored complex with Ecad. Therefore, one may expect that meaningful biological competition may still occur, especially at high local concentrations of punicalagin alone or as part of phytocomplexes/plant extracts, that could act synergistically. In this context, these results, together with the experimental data available for the anti-adhesive activity of punicalagin [31,32,37,38], should encourage future in vitro studies addressing the concentration dependence of the anti-adhesive activity of punicalagin, as well as biophysical studies focused on the structural and energetic validation of the interaction with punicalagin.

On the other hand, CNP0154950 and CNP0101638 exhibit stable binding to clusters 2 and 3 (Table S1, Supplementary Materials), thus mimicking the interaction found for punicalagin with similar or slightly lower interaction energies. Although experimental validation is still required to confirm the anti-adhesive action of these two compounds, the results obtained for punicalagin support the application of the computational pipeline to exploring novel natural compounds able to prevent the recognition between InIA and Ecad as a mechanism of action against listeriosis.

### 2.4. Possible Practical Application of the Outcomes in the Food Sector

Nowadays, almost all the LM human infections are foodborne; thus, action in the food sector to either prevent or counteract LM spread is advisable. Moreover, both consumers and food stakeholders are increasingly becoming aware of the importance of using compounds of natural origin with antimicrobial properties in the food chain. First, the abuse of antibiotics over the years has promoted the emergence of resistant pathogenic strains and pushed the scientific community to find alternatives to so-called conventional methods and legacy antibiotics [46]. The second reason is related to consumers’ growing trend to prefer foods with additives from natural sources over those produced by industries [47]. Moreover, a wealth of natural compounds, besides the well-known “natural” antibiotics described so far, have already been proved to have good antibacterial properties against a broad range of foodborne pathogens, including LM [48,49,50,51]. In this context, natural compounds may be a promising strategy to better comply with the current scenario.

CNP0154950 and CNP0101638 belong to two different classes of molecules of natural origin: gallotannins and oleanane triterpenoids. Noteworthily, both classes of compounds have already been proven to have antibacterial properties, and specifically anti-LM activity, including the inhibition of biofilm formation and the growth inhibition or neutralization of bacterial toxins [51,52,53,54,55,56]. Of note, some of the available studies highlighted the capabilities of these classes of compounds to interfere with adhesion and invasion, both features known to be influenced by InlA [30,31].

In this regard, a wealth of studies also described the extractability of gallotannins from many different sources, including some considered for or deriving from the food production chain [57], further supporting the relevance to the sake of the study. Concerning oleanane triterpenoids, oleanolic acids are widely diffused in plants, with many high-content species entering the food production chain a long time ago, like *Olea europaea* L., and for whom the extraction of oleanolic acid fractions is reasonable to consider [58]. Indeed, moving to a real-world scenario, the applicability of the identified compounds to enhance the food safety of certain products could exploit the pytocomplexes included in plant extracts [59]. This aligns with many of the previously reported studies, as the isolation of individual compounds would certainly be more challenging, both time- and money-wise. Furthermore, phytocomplexes might better meet “clean label” trends, not underestimating the consumer’s preferences [60]. However, their toxicity, their possible combined effect with other compounds, and their safety profile more generally, including any drug–drug interactions, should be thoroughly investigated prior to their practical applications. Lastly, it should always be considered how these compounds could possibly lead to unwanted taste/aroma when added to certain food matrices as additives, rather than being used as sanitizers to clean out the producing environment or as food contact materials following the trend of active packaging, as reported by Arruda and co-workers [61]. For this reason, further research should also be conducted to find the best threshold to achieve the desired antibacterial activity without altering the sensory characteristics.

The findings presented here, other than expanding the knowledge on punicalagin, might provide a mechanistical explanation of the mode of action of the currently unknown active molecules present in natural extracts proven to be effective against LM invasion and/or adhesion. However, such natural compounds are known for their promiscuous binding profiles [62,63], and while this could contribute to their broader antimicrobial effect it might pose potential risks worthy of further investigations. As a general remark, this fully computational study, crucial for exploratory purposes, would benefit from experimental evidence. Among these follow-ups—which would deserve dedicated studies—isothermal titration calorimetry could be employed to experimentally assess the binding affinity of punicalagin and the two newly proposed natural compounds (i.e., CNP0154950 and CNP0101638) to InlA. In addition, cell invasion assays and/or anti-adhesion activity assays could also be used to verify whether these compounds specifically inhibit LM adhesion, invasion, or both. To sum up, this study provided a solid foundation for further dedicated in vitro and in vivo analyses to test their actual usability, alongside testing their safe use in the food industry, possibly lowering LM diffusion, infection, and, consequently, burden of disease.

## 3. Materials and Methods

### 3.1. Data Source

#### 3.1.1. Protein Structure

The 3D structure of the LM InlA–human Ecad complex was obtained from the Protein Data Bank (PDB; https://www.rcsb.org (accessed on 4 of March 2024)) [64], under the PDB ID 1O6S [20]. The structure was solved at 1.80 Å through X-ray diffraction, enabling a precise identification of the residues that shape the interface between InlA and Ecad. The InlA–murine Ecad complex was obtained by mutating the 1O6S human Ecad residues to the murine ones, reported in Table 1. This was performed by using the PyMol (v. 2.3) Wizard mutagenesis tool. Of note, the residue numbering adopted alongside the manuscript was consistent with the one associated with the crystal structure with the PDB ID 1O6S.

#### 3.1.2. Natural Compounds Library

The chemical structures of natural compounds were retrieved from three publicly available databases: COCONUT (https://coconut.naturalproducts.net/ (accessed on 20 March 2024)) [39], LOTUS (https://lotus.naturalproducts.net/ (accessed on 20 March 2024)) [40], and FOODB (https://foodb.ca/ (accessed on 20 March 2024)). All molecules were defined by using their SMILES code.

The three databases in the SMILES format were merged and duplicate entries were removed, leading to a virtual library of ~600,000 non-redundant compounds. Subsequently, they were filtered by using an ad hoc python script mainly based on the RDKit toolkit implemented for python to exclude molecules not meaningful for the study, mainly in terms of molecular size and polarity, considering their possible use and mode of action in aqueous environments. The following criteria were followed: logP (−1 ≤ logP ≤ 3), rotatable bonds (rotatable bonds ≤ 5), molecular weight (650 ≤ molecular weight ≤ 1100), and “molecular length”, defined as the highest distance between two atoms belonging to the same molecule (molecular length ≤ 15 Å). The first two parameters, logP and rotatable bonds, were set to obtain molecules possibly acting in a water-based environment without excessive flexibility and thus with reduced conformational penalty to enable the interaction with InIA. The last two size-driven parameters were set to filter only molecules able to interact with the cluster of residues identified in the inner surface of InlA (see Section 3.2).

The filtering procedure led to a reduction by two orders of magnitude of the considered dataset, from over 600,000 starting compounds to 4986 compounds, including punicalagin (CID 16129719). The LigPrep module of MAESTRO (v. 3.4) [65] was used to prepare the 3D structure library of the compounds. Specifically, it was set to generate up to 8 stereoisomers and 6 tautomers per compound, considering a protonation state at a pH of 7.4. This generated a final library of more than 20,000 chemical species (on average, 5 chemical species per compound).

### 3.2. Molecular Dynamics Simulations

Molecular dynamics (MD) simulations were conducted by using the AMBER20 package [66]. InlA and Ecad were treated by adopting the ff19SB forcefield [67]. The ligands were parametrized with the GAFF forcefield [68], and partial atomic charges were derived following the RESP protocol [69] at the B3LYP/6-31G(d) level of theory using Gaussian16 [70]. All the simulated systems were neutralized (adding Na^+^ and Cl^−^ at 0.15 M) and embedded within an octahedral box of OPC3 water molecules (roughly 45,000 water molecules/system) [71].

Each system was energetically minimized in three steps involving (i) hydrogen atoms, (ii) water molecules and ions, and (iii) the whole system. After minimization, each system was equilibrated via thermalization to 300 K (within 600 ps, 700 ps, or 800 ps, depending on the replica) and pressurization to 1 bar (within 1 ns). Prior to MD production, an equilibration in NVT ensemble was run for 1 ns. Regarding MD production, 3 independent MD simulations of 1 μs each were run for the InlA–E-cad complex (alternatively with human or murine Ecad) to check the consistency of the results. On the other hand, MD simulations for the InlA–ligand systems were run for 400 ns. Regarding the latter, a restraint of 5.0 kcal/mol/Å^2^ for the ligand was kept during the equilibration phase, and subsequently reduced to 2.5 kcal/mol/Å^2^ during the first 10 ns of MD production, to avoid artefactual changes due to thermalization of the simulated system. For those ligands that showed a stable interaction with InIA along the trajectory, two additional MD simulations were run to check the structural integrity of the InlA–ligand complex.

A per-residue free energy decomposition analysis was conducted, exploiting the MMPBSA.py [72] available in the AmberTools23 package [66], to study the differences in terms of energy contribution to the InlA–Ecad complex of the eleven distinct residues between murine and human Ecad. Moreover, MMPBSA.py was also used to compute the interaction free energy between InlA and Ecad, as well as between InlA, punicalagin, and the two most promising ligands (i.e., CNP0154950 and CNP0101638), which exhibited a stable binding mode along the MD simulations of the InIA–ligand complex.

### 3.3. Docking-Based Screening

The virtual screening of the filtered dataset of natural compounds was completed through molecular docking computations, which provided a plausible binding architecture at the InlA surface. It was conducted using the Glide docking program (version 91117, Schrödinger Release 2021-3: Schrödinger, LLC, New York, NY, USA, 2021) [73,74]. A semi-flexible procedure where the receptor (InlA) was rigid, and the ligand was fully flexible, was adopted. The box considered by Glide to perform the docking was set to encompass clusters 1, 2, and 3 of the InlA–Ecad complex (30 Å × 20 Å × 20 Å), previously identified through MD simulations (see Section 3.2.).

The first round of docking-based screening was performed using the HTVS mode, which is the most computationally efficient. Subsequently, the top-ranked 4000 chemical species—no threshold was a priori set—were docked using the SP scoring function, which includes a more elaborate formalism, allowing final torsional refinement and sampling. All other parameters were kept as per default settings, allowing the generation of 10 poses per ligand (*POSES_PER_LIG 10*) and ring conformation energy below 2.5 kcal/mol (*RINGCONFCUT 2.500000*). The best poses of the top-ranked 500 compounds were visually inspected. Together with punicalagin, the binding mode of the selected compounds (CNP0154950, CNP0095328, CNP0101638, and CNP0094932) was finally refined using MD simulations (see above). Although the computational docking procedure targeting InlA could not be explicitly validated due to the lack of known small-molecule binders, the recovery of punicalagin within the top-ranked compounds gives confidence to the docking protocol.

## 4. Conclusions

In the present study, we proposed a computational pipeline to investigate the InlA–Ecad interaction to identify natural compounds capable of interfering with LM adhesion, a key step in the infection process. The analysis of MD simulations allowed for the identification of three main interaction clusters at the InlA–Ecad interface, which were prioritized as putative anchoring regions for inhibitory compounds. This strategy successfully retrieved punicalagin, whose stable binding was supported by multiple interactions within clusters 2 and 3. Two additional phenolic compounds exhibiting a similar interaction pattern were recorded as well. These findings provide a mechanistic rationale supporting the anti-adhesive potential of these compounds and pave the way for further dedicated studies to validate their application in mitigating LM virulence/spread, particularly in the context of food safety. Last, the use of these compounds may offer a cost-effective solution in line with clean label trends. However, their safety, off-target interactions, and impact on sensory profiles must be evaluated. Moreover, while punicalagin and the two newly proposed compounds showed promising binding to InlA, their bioavailability/pharmacokinetics should require a careful analysis since the structural complexity may affect properties such as intestinal absorption when adopted as food additives [75], warranting further investigation into their in vivo behavior and metabolism.

## Figures and Tables

**Table 1:** Per-residue free energy decomposition of the 11 distinct residues between human and murine Ecad. The position reported here is relative to the crystal structure 1O6S [20].

E-Cadherin	Residue	Position	Average (kcal/mol)
Human	**PRO**	**16**	**−7.0**
	LYS	28	0.6
	GLY	32	0.1
	THR	45	0.3
	**GLU**	**64**	**−3.9**
	ARG	70	1.3
	THR	73	0.2
	THR	75	0.2
	PHE	77	0.1
	ASN	86	0.0
	LEU	95	0.2
	Total contribution		**−7.9**
Murine	**GLU**	**16**	**−5.2**
	ARG	28	0.3
	THR	32	0.1
	LYS	45	0.3
	**GLN**	**64**	**−1.9**
	ALA	70	0.1
	LYS	73	0.5
	ILE	75	0.1
	TYR	77	0.1
	GLU	86	0.1
	VAL	95	0.3
	Total contribution		**−5.1**

## Data Availability

The datasets presented in this article are not readily available because they are part of the PhD thesis of Pedroni, which is still under embargo. Requests to access the datasets should be directed to the corresponding author, Luca Dellafiora (luca.dellafiora@unipr.it).

## Supplementary Materials

The following supporting information can be downloaded at: https://www.mdpi.com/article/10.3390/ijms26157327/s1.

## Abbreviations

The following abbreviations are used in this manuscript:

CSA	Contact Surface Area
Ecad	E-cadherin
InlA	Internalin A
LLR	Leucine-Rich Repeat
LM	*Listeria monocytogenes*
PDB	Protein Data Bank
MD	Molecular Dynamics
RMSD	Root Mean Squared Deviation
