# Supplementary material for: Modeling the Anti-Adhesive Role of Punicalagin Against Listeria Monocytogenes from the Analysis of the Interaction Between Internalin A and E-Cadherin
Source: Int J Mol Sci. 2025 Jul 29;26(15):7327. doi: 10.3390/ijms26157327 (PMC12347816; doi:10.3390/ijms26157327)
Supplement: Supplementary file 1 [file ijms-26-07327-s001.zip › ijms-3738818-supplementary.pdf]

International Journal of *Molecular Sciences*

Supplementary Material

Modeling the anti-adhesive role of punicalagin against *Listeria monocytogenes* from the analysis of the interaction between internalin A and E-cadherin

Lorenzo Pedroni [1], Sergio Ghidini [2], Javier Vázquez [3,4], Francisco Javier Luque [5] and Luca Dellafiora [1,*]

[1] Department of Food and Drug, University of Parma, 43124 Parma, Italy
[2] Department of Veterinary Medicine and Animal Sciences, University of Milan, 20122 Milan, Italy
[3] Departament de Nutrició, Ciències de l'Alimentació i Gastronomia, Facultat de Farmàcia i Ciències de l'Alimentació, Institut de Biomedicina (IBUB), Universitat de Barcelona, 08021 Santa Coloma de Gramenet, Spain
[4] Pharmacelera, Parc Científic de Barcelona (PCB), Baldiri Reixac 4-8, 08028 Barcelona, Spain
[5] Departament de Nutrició, Ciències de l'Alimentació i Gastronomia, Facultat de Farmàcia i Ciències de l'Alimentació, Institut de Química Teòrica i Computacional (IQTCUB) and Institut de Biomedicina (IBUB), Universitat de Barcelona, 08021 Santa Coloma de Gramenet, Spain
* Correspondence: luca.dellafiora@unipr.it; Tel.: +39 0521 906070

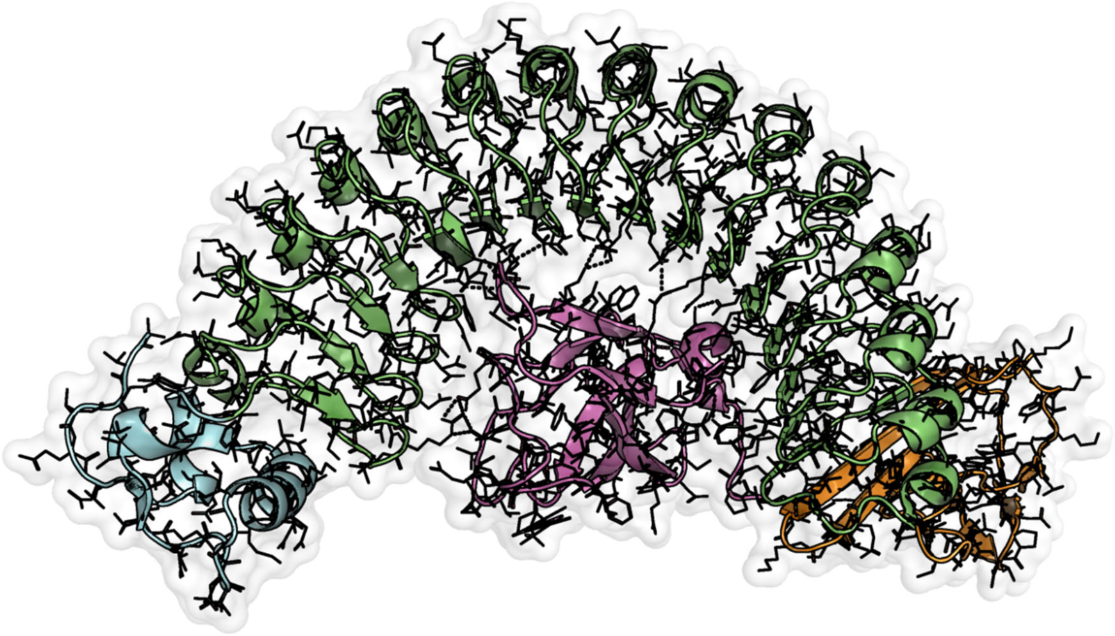

Figure S1. Internalin A – human E-cadherin crystallographic structure (PDB ID 1O6S). Both InlA and Ecad are represented as cartoons, with residues represented as lines and with white transparent surface. The Ecad is magenta while InlA has a cyan C-terminal, a green LRR domain and an orange IR domain.

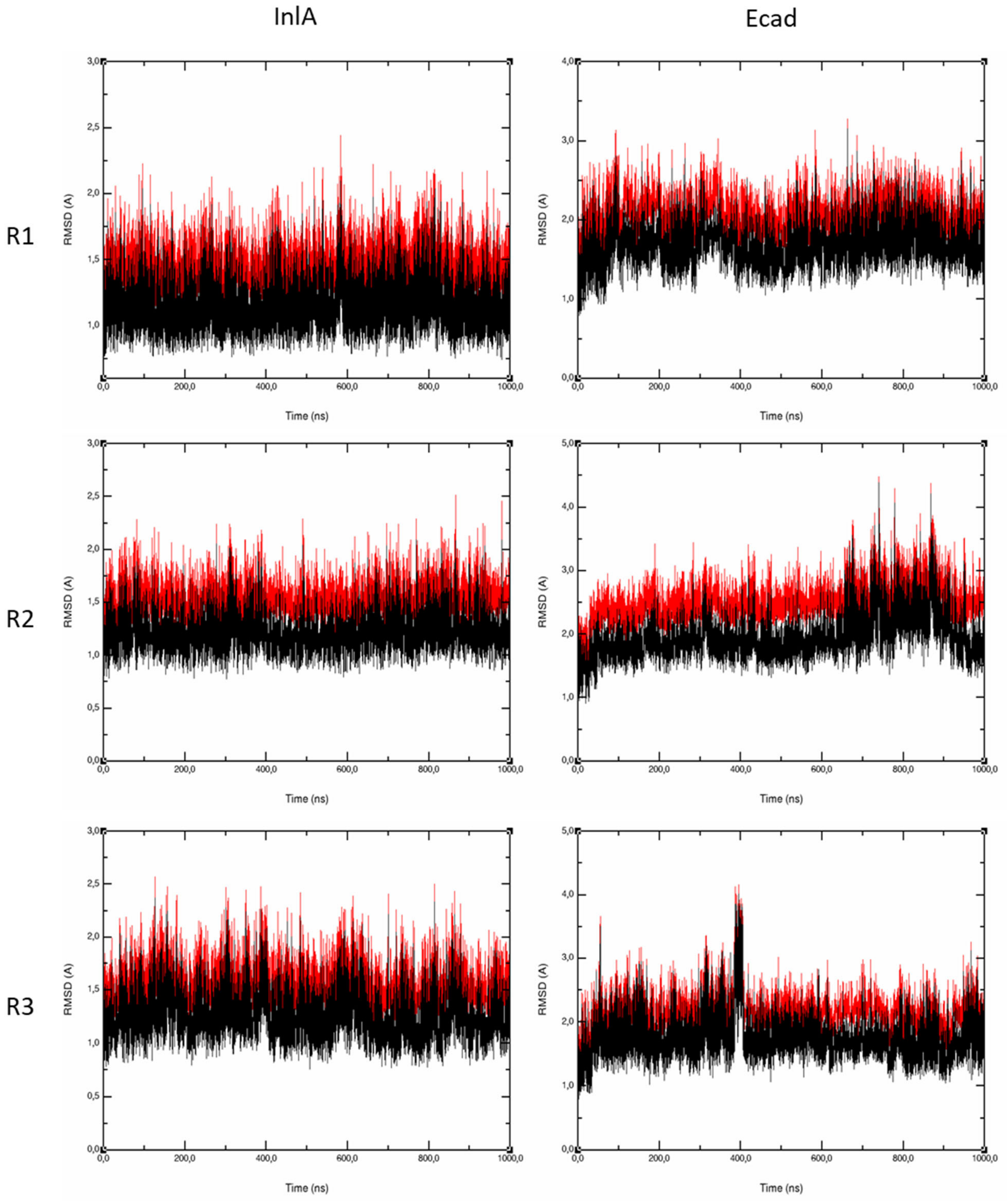

Figure S2. Time evolution of the RMSD (Å) of InlA, on the left, and Ecad, on the right, over the three 1 μs long MD simulations replicas. The red line represents the RMSD computed over all the protein's heavy atoms while the black one considering only the backbone.

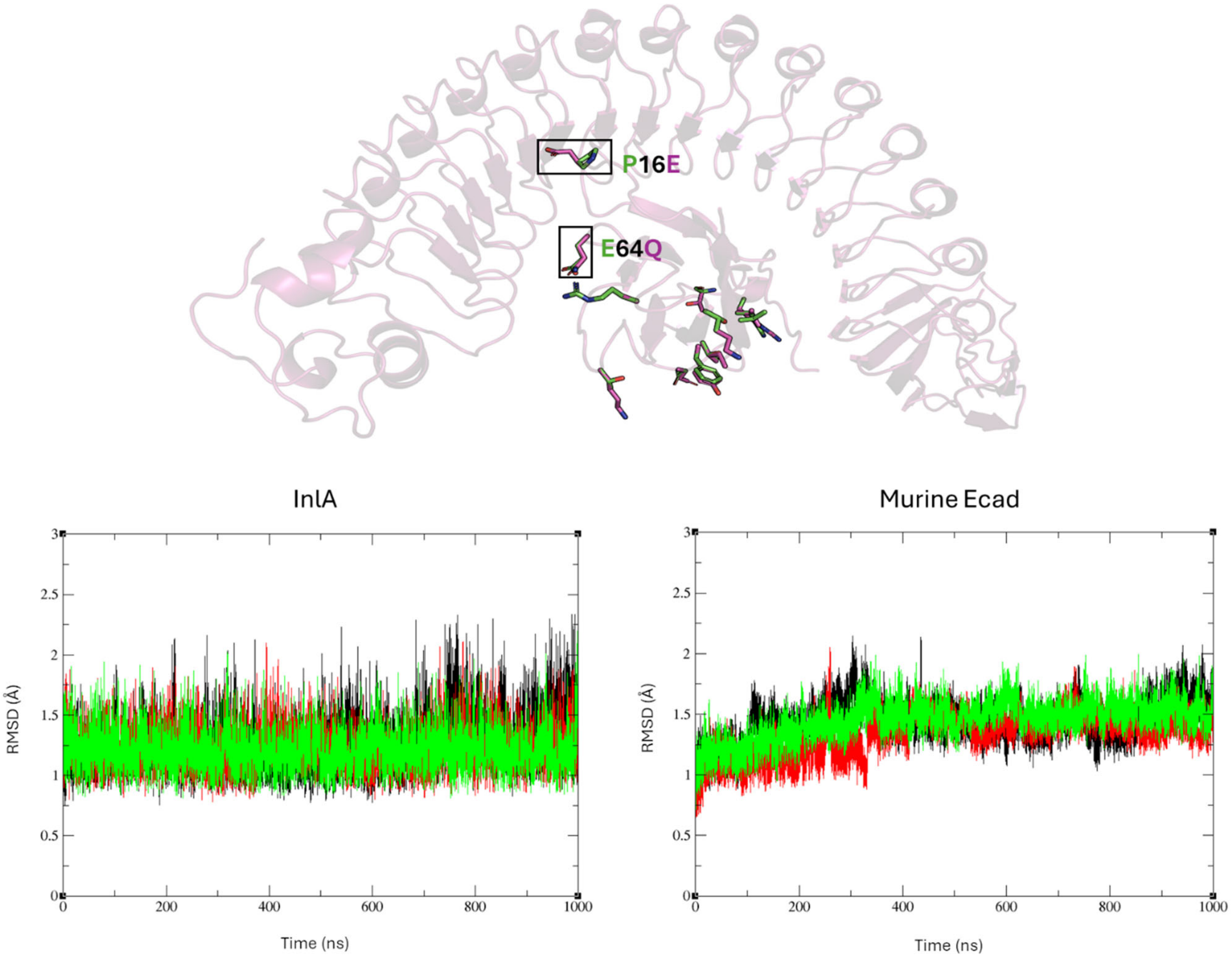

Figure S3. In the upper part, InlA – murine Ecad represented as magenta transparent cartoon. Human residues represented as green sticks while murine residues represented as magenta sticks with a focus on the two mutations considered critical, i.e. P16E and E64Q (residue numbering as per PDB ID 1O6S). In the lower part, InlA's and murine Ecad's backbone RMSD (Å) profile over the three 1 μs long MD simulations replicas (black, red and green). While InlA stability can be appreciated since the beginning of the simulation, murine Ecad stabilizes after a first settling. This was expected since the starting pose was based on PDB ID 1O6S having eleven different residues.

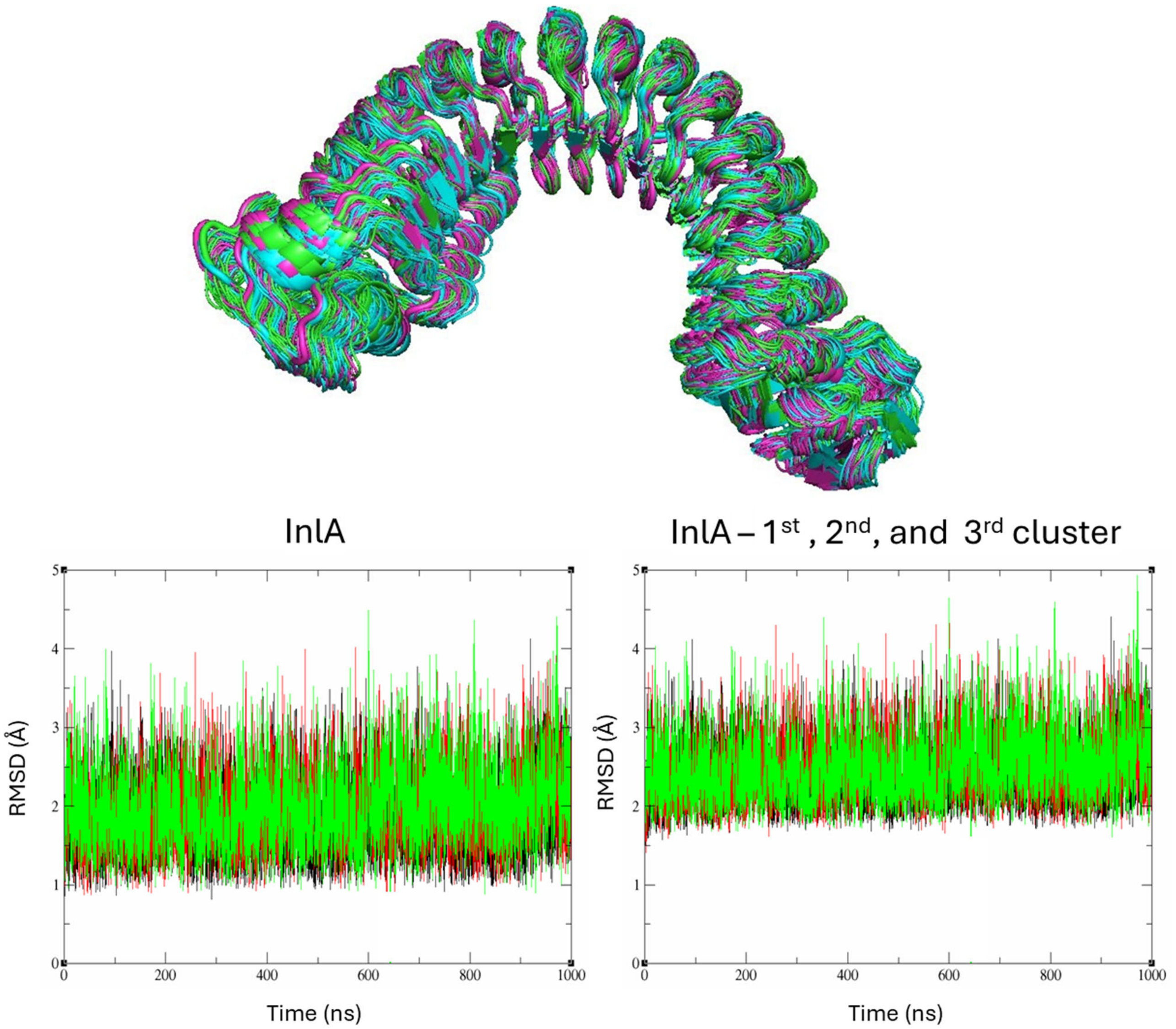

Figure S4. On the top, collection of 50 snapshots (1 every 20 ns) of the 1 μs long MD simulations replicas of InlA. The protein is represented as a cartoon differently coloured depending on the replica (green, magenta and cyan). It can be noticed how most of the mobility is given by the N- and C-terminal region while the clamp-alike structure over the LRR domain is maintained. On the bottom, InlA's backbone RMSD (Å) profile on the left over the three 1 μs long MD simulations replicas (black, red and green) and on the right, RMSD of the backbone of residues constituting the first, second, and third cluster over the three 1 μs long MD simulations replicas (black, red and green). Both RMSDs showed a comparable stability to InlA when in complex with Ecad.

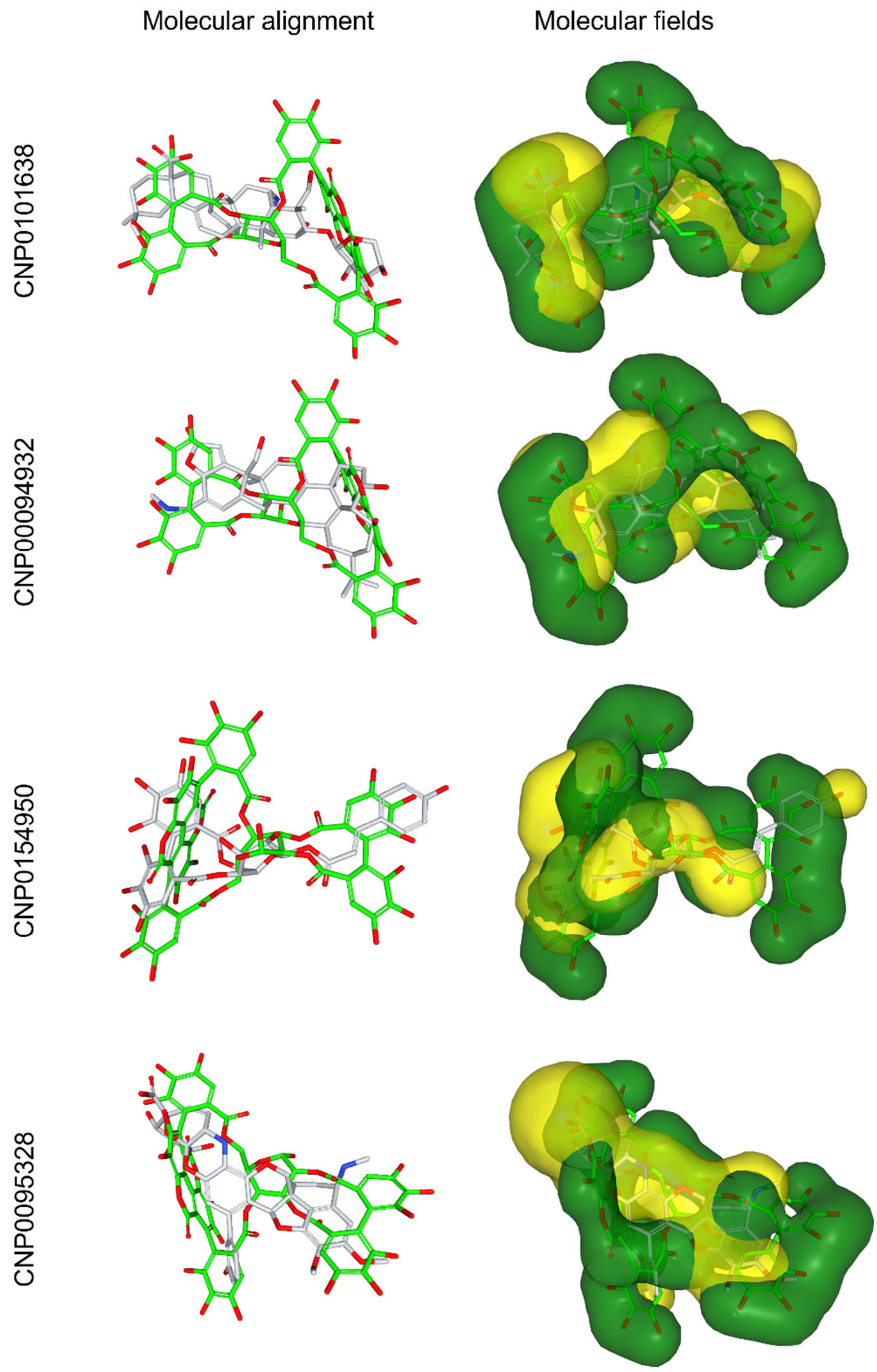

Figure S5. (Left) Pairwise overlay of punicalagin (green sticks) and compounds CNP0101638, CNP00094932, CNP0154950 and CNP0095328 (grey sticks) obtained with PharmScreen. (Right) Isocontour representation of the molecular fields obtained from the electrostatic contribution to hydrophobic/philic HyPhar descriptors for punicalagin (dark green) and the four phenolic compounds (yellow).

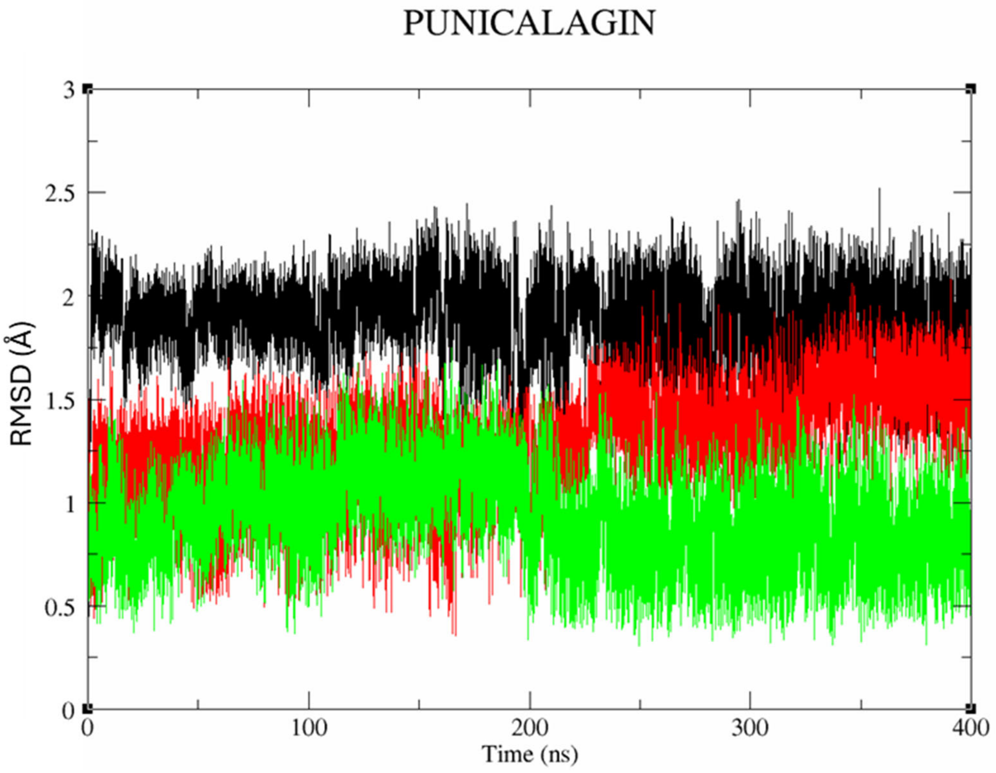

Figure S6. Time evolution of RMSD (Å) of punicalagin over the three replicas (black, red, and green). It can be seen how the trends are similar in all the three replicas apart from slight readjustments reaching a stability.

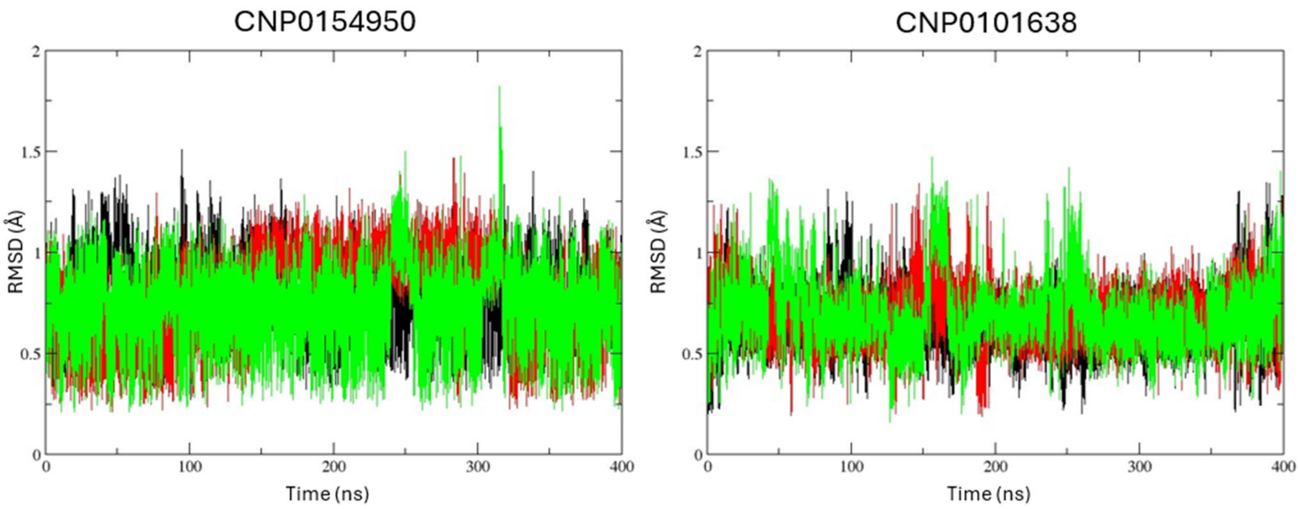

Figure S7. Time evolution of RMSD (Å) of CNP0154950 (on the left) and CNP0101638 (on the right) over the three replicas (black, red, and green). It can be seen how the trends are similar in all the three replicas apart from slight readjustments.

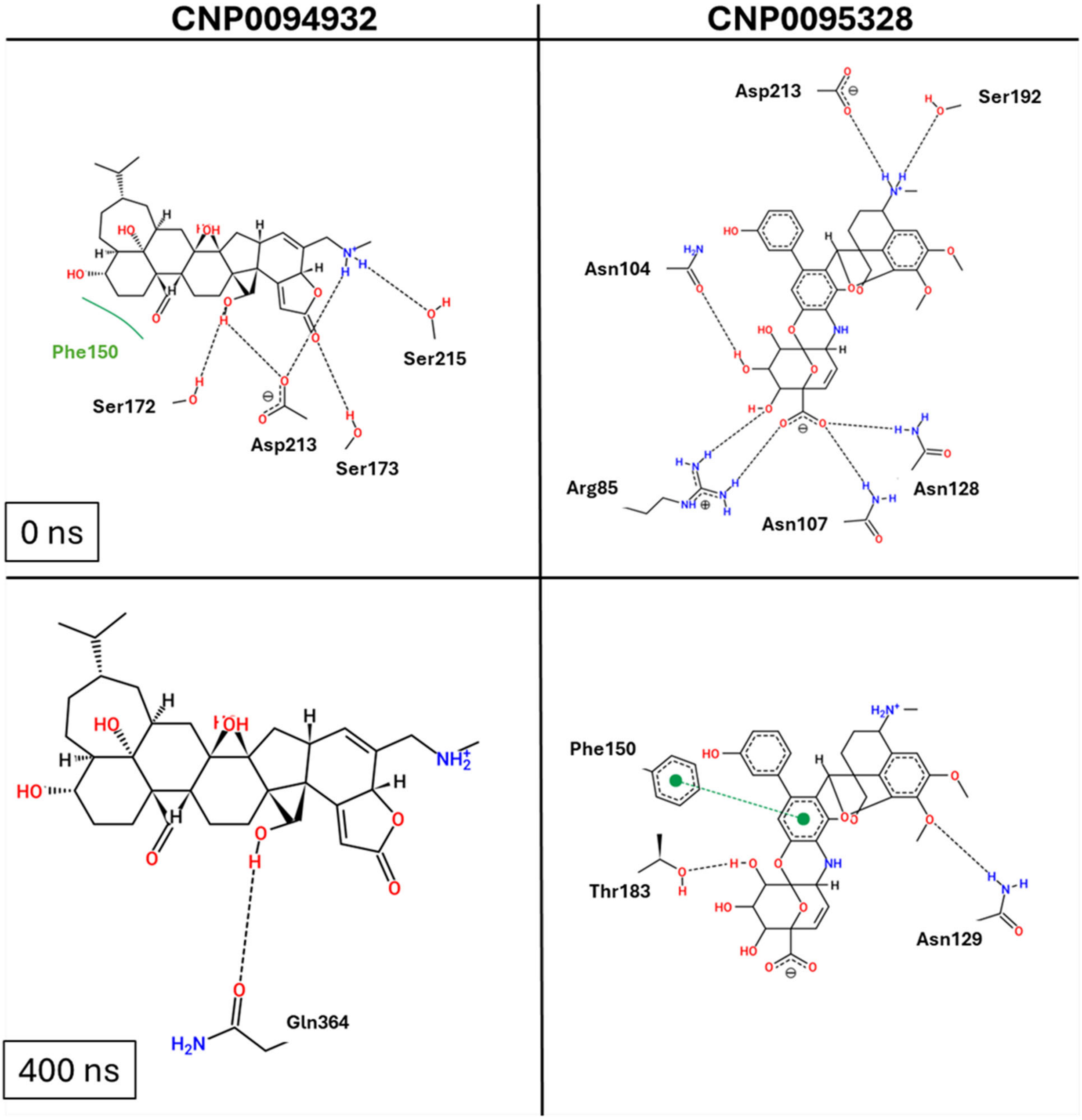

Figure S8. 2D diagram of the main contacts between CNP0094932 (left) and CNP0095328 (right) and InlA within the one MD replica at time 0 ns (above) and 400 ns (below). Interaction types are color-coded as it follows: black dashed lines for polar interactions (hydrogen bonds, salt bridges), green curved lines represent hydrophobic contacts, and green dots connected by green dashed lines report pi-pi stacking interactions.

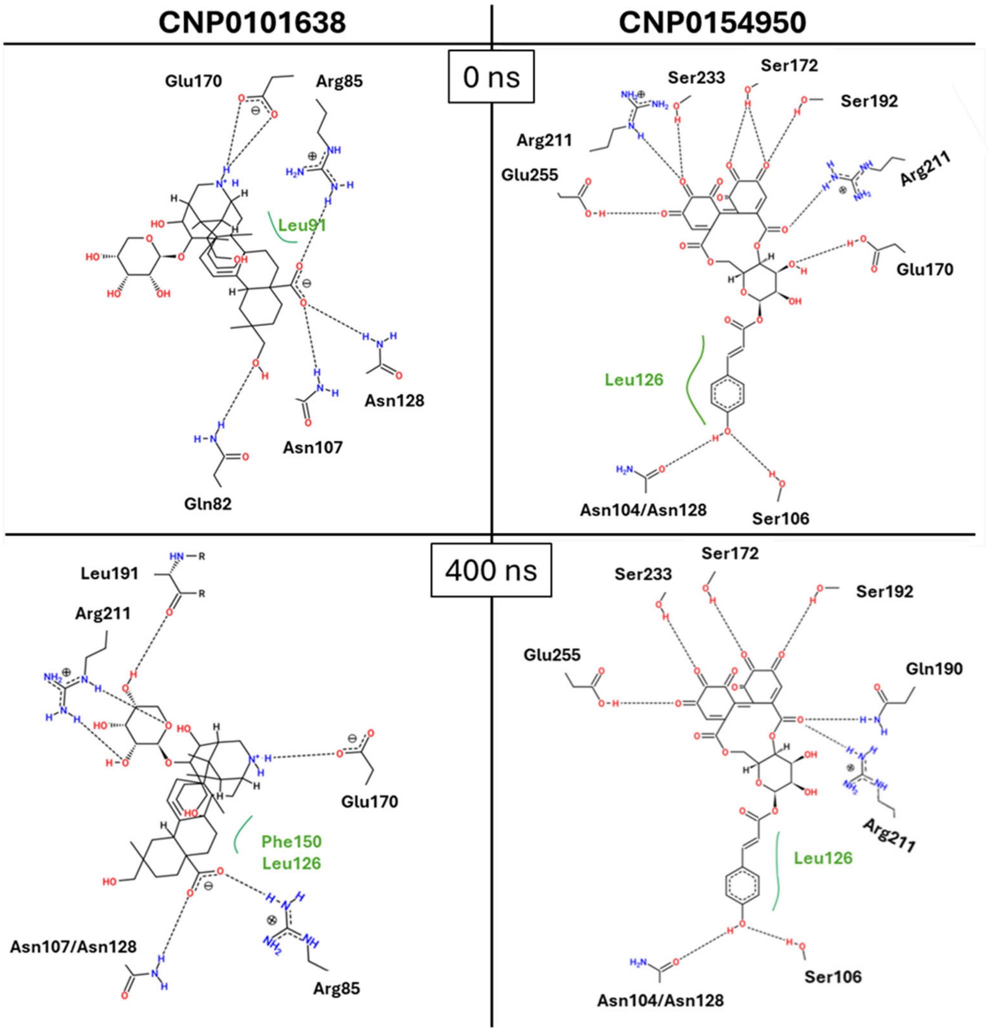

Figure S9. 2D diagram of the average contacts between CNP0101638-InlA (left) and CNP0154950-InlA (right) within the three MD independet replicas at time 0 ns (above) and 400 ns (below). Interaction types are color-coded as follows: black dashed lines for polar interactions (hydrogen bonds, salt bridges), green curved lines represent hydrophobic contacts.

Table S1. Residues contacted by punicalagin, CNP0101638, and CNP0154940

**Ligand**	**Key-residues contacted**
Punicalagin	Q102, D124, R168, E170, Q190, **R211**, **S233**, **E255**
CNP0101638	R85, **N107**, L126, **N128**, F150, **E170**, L191, **R211**
CNP0154950	N104, S106, L126, **N128**, **E170**, S172, Q190, **S192**, **R211**, **D213**, S233, **E255**

Note: *residues considered to be involved in key interactions are reported in bold.*
